# Comorbid atypical autistic traits as a potential risk factor for suicide attempts among adult depressed patients: a case–control study

**DOI:** 10.1186/s12991-014-0033-z

**Published:** 2014-10-16

**Authors:** Kiyoharu Takara, Tsuyoshi Kondo

**Affiliations:** Department of Neuropsychiatry, Graduate School of Medicine, University of the Ryukyus, 207 Uehara, Nishihara, Okinawa 903-0215 Japan

**Keywords:** Autism spectrum disorder, Adult, Atypical, Depressive episode, Suicide attempt, Risk factor, Pervasive developmental disorder-not otherwise specified, Suicide methods

## Abstract

**Background:**

The present study aims to examine if autism spectrum disorder (ASD) is a risk factor for suicide attempts among adult depressed patients and to elucidate the characteristics of suicide attempts in adult depressed patients with ASD.

**Methods:**

We conducted a case–control study. Subjects consisted of 336 retrospectively recruited first-time visit patients to our outpatient clinic with a current major depressive episode; 31 of the 336 patients had attempted suicide. The demographic backgrounds (i.e., age, gender, personal/family history of suicidality); specific psychopathology like bipolarity, agitation, and psychotic features; and comorbidity such as physical diseases, alcohol abuse, cluster B personality disorder, and ASD including pervasive developmental disorder not otherwise specified (PDD-NOS) were examined as potential risk factors for suicide attempts. We compared these variables between the suicide attempters and non-attempters. In addition, we compared suicide attempters to non-attempters within the ASD group and non-ASD group. Binary logistic regression analysis was performed using the significant independent variables from the comparisons between the suicide attempters and non-attempters, and the odds ratios (OR) and 95% confidence intervals (CI) were calculated.

**Results:**

Logistic regression analysis demonstrated that agitation during a depressive episode (OR = 7.15, 95% CI = 2.88–17.74), past suicidal behaviors (OR = 4.32, 95% CI =1.70–10.98), and comorbid PDD-NOS (OR = 4.04, 95% CI = 1.20–13.54) were significantly associated with suicide attempts. The most prevalent suicidal method was drug overdose (59.1%) among non-ASD attempters while hanging was the most prevalent (44.4%) in ASD attempters.

**Conclusions:**

Depressed adults with comorbid atypical autistic traits are at higher risk for suicide attempts and may engage in methods that are more lethal.

## Background

Autism spectrum disorders (ASDs) are neurodevelopmental disorders characterized by deficits in social communication and social interaction and the presence of restricted, repetitive behaviors. ASDs include three of five disorders known as pervasive developmental disorders (PDDs) in the DSM-IV-TR [1]. These consist of autistic disorder, Asperger’s disorder (AS), and pervasive developmental disorder not otherwise specified (PDD-NOS).

Recently, the prevalence of ASD in adults was estimated to be 0.98% [2]. Thus, ASD has nowadays become a more common disorder than previously recognized. Besides, it has been consistently pointed out that mood disorders are the most common lifetime comorbidity (53%–70%) among ASD adults [3-5]. In addition, mood disorders are generally accompanied by suicide risk [6].

Several studies have consistently reported that many individuals with AS experience suicide-related behaviors [7-10]. McDermott et al. reported that the relative rate (RR) of self-inflicted injury or suicide attempts was much higher among children with autistic disorder compared to those without the disability in emergency departments and inpatient admissions (RR = 7.62, 95% confidence interval = 1.65–35.21) [11]. Mayes et al. also showed that the percentage of children with autistic disorder who had suicide ideations or attempts was 28 times greater than that of typically developed children (13.8% vs. 0.5%) [12]. These studies indicate that suffering from severe forms of ASD can be a risk factor for suicide attempts.

Meanwhile, only a few studies have examined the nature of suicide-related behaviors in individuals with ASD, comprehensively including severe to mild or typical to atypical psychopathology [13,14], although psychiatrists may encounter depressed individuals comorbid with various subtypes of ASD who may be at high risk of suicidality. Therefore, the present study aims to examine if ASD is one of the important risk factors for suicide attempts among adult depressed patients in clinical settings and to elucidate the characteristics of suicide attempts in individuals with ASD.

## Methods

### Subjects

We conducted a case–control study in a retrospective manner to elucidate the relation between autistic traits and suicide attempts. Subjects were adult outpatients, who had initially visited our clinic from January 2009 to December 2012 and suffered from a current major depressive episode but without apparent intellectual problems. Subjects with a high-level education (regular high school education or higher) were automatically regarded as not having apparent intellectual problems. In doubtful cases, we estimated intelligence by using the Japanese version of the Adult Reading Test (JART) [15] as a simple estimation of intelligence. The JART consists of 50 Japanese irregular words to estimate pre-morbid or prior intellectual ability. The original version of the National Adult Reading Test developed by Nelson and Wilson [16] was proven to be a robust but adequate predictor of IQ scores.

Initially, 381 patients were enrolled in the present study, and their final diagnosis was reconfirmed in January 2014. Among the 381 patients, 45 did not have precise descriptions of their background or personal history (e.g., insufficient developmental record and family history) and thereafter were excluded from the study as missing data. We finally extracted 336 patients with mood disorders, whose mean age (±standard deviation) was 41.2 (±14.3) years, and the proportion of females was 62%. Of the 336 patients, 206 were diagnosed with major depressive disorder (61%) and 130 with bipolar disorders (39%). The sample included 31 patients visiting after suicide attempts (9.2%) while the remaining 305 cases had not attempted suicide. For the group of suicide attempters, the methods of suicide attempts were recorded as necessary data information for the analyses.

### Screening and subjective awareness of autistic traits

The autistic traits in ASD subjects were quantitatively assessed by using the Japanese version [17] of the Autism-Spectrum Quotient (AQ) [18], which is a 50-item self-administered questionnaire for adults with normal intelligence. The total AQ score ranges from 0 to 50. Although the scoring of AQ can be affected by patients’ self-assessment capability for their autistic traits, a higher AQ score generally indicates a higher autistic tendency.

### Diagnosis of ASD

The Structured Clinical Interview for DSM-IV Axis I Disorders (SCID-I) [19], Axis II personality disorders (SCID-II) [20], and the DSM-IV-TR were used for the diagnosis of mood disorders, various subtypes of ASD, and other psychiatric comorbidity. Developmental history for the ASD diagnosis was confirmed by the patients’ parents interviewed by the psychiatrists, who were qualified as certified supervising psychiatrist by the Japanese Board of Psychiatry and who were in charge of the outpatient clinic for both child-adolescent and adult psychiatry. Developmental records (maternal and children health handbooks, school reports in primary and middle school) were obtained from the parents as references. In those cases where parents could not visit and be interviewed face-to-face, we collected the developmental information by telephone interview after approval from the patients and their parents.

### Variables investigated

Possible risk factors for suicide attempts were selected according to empirical findings and previous literature, i.e., patients’ backgrounds (age, gender, educational level, marital status, employment, and living conditions), past suicidal behaviors, family history (psychiatric disorders and suicide deaths), specific psychopathology of depression (bipolarity, agitation, and psychotic features), comorbidity (physical diseases, alcohol abuse, cluster B personality disorders, and ASD), and treatment at the first visit (untreated vs. treated, drug treatments such as antidepressants, anxiolytics, antipsychotics, and mood stabilizers).

The rationale for selecting these 16 variables was based on previous findings that reported the following risk factors for suicide attempts or suicide, i.e., younger age [21,22], female gender [21,22], low education level [21,22], unmarried status [21,22], unemployment [22,23], living alone [24,25], past suicidal behaviors [24,26], family history of psychiatric disorders [27,28] and suicide deaths [27], bipolarity [29,30], agitation during a depressive episode [31,32], psychotic features [33,34], physical diseases [35,36], alcohol abuse [24,37], cluster B personality disorders [38,39], and ASD.

Past suicidal behaviors were defined as past histories of overdose, deliberate self-injury, hanging, or jumping. Agitation was assessed according to the criteria B of agitated depression, which was defined by Koukopoulos and Koukopoulos [40] and consists of at least two of the following symptoms: 1) motor agitation, 2) psychic agitation or intense inner tension, and 3) racing or crowded thoughts. Psychotic features during depressive episodes were evaluated based on the DSM-IV-TR criteria. Cluster B personality disorders were assessed by using the SCID-II.

### Statistical analyses

Comparisons were made between suicide attempters and non-attempters among all subjects (Table 1) and between suicide attempters and non-attempters within the ASD or non-ASD group (Table 2) by using a Student’s *t*-test (for continuous variables) or Fischer’s exact test (for categorical variables). Binary logistic regression analysis (Table 3) examined the individual effect of each risk factor on suicide attempts by using six significant variables from Table 1. As for the age factor, the optimal cutoff (under 29 years vs. 29 or over) was calculated as the best criterion that divided the ASD group from the non-ASD group by using the Youden index (i.e., sensitivity + specificity −1) [41]. Finally, the age factor was changed from a continuous to a categorical variable for the binary logistic regression analysis.

**Table 1:** **Comparisons between suicide attempters and non**-**attempters among 336 depressed adults**

	**Suicide attempters** **(** ***n*** **= 31)**	**Suicide non**-**attempters** **(** ***n*** **= 305** **)**	***p***
Age (year) mean (SD)	35.6 (13.5)	41.8 (14.3)	.02
Range	19–62	18–83	
	*n* (%)	*n* (%)	
Under 29 years old	15 (48)	59 (19)	<.01
Female gender	20 (65)	188 (62)	.85
Already treated at the first visit	23 (74)	190 (62)	.24
Drug treatments at the first visit	19 (61)	171 (56)	.70
Anxiolytics	10 (32)	109 (36)	.84
Antidepressants	11 (35)	100 (33)	.84
Mood stabilizers	6 (19)	40 (13)	.41
Antipsychotics	5 (16)	43 (14)	.79
Educational level			
University or upper	9 (29)	136 (45)	.13
High school or lower	22 (71)	169 (55)	
Marital status			
Married	15 (48)	172 (56)	.45
Unmarried, divorced, or widowed	16 (52)	133 (44)	
Employment			
Employed, homemaker, undergraduate	20 (65)	206 (67)	.84
Unemployed or retired	11 (35)	99 (33)	
Living alone	2 (6)	65 (21)	.06
Past suicidal behaviors	16 (52)	41 (13)	<.01
Family history of psychiatric disorders	9 (29)	77 (25)	.67
Family history of suicide	6 (20)	20 (7)	.02
Bipolarity	13 (42)	117 (38)	.70
Agitation	18 (58)	37 (12)	<.01
Psychotic features	4 (13)	17 (6)	.12
Physical diseases	3 (10)	49 (16)	.44
Alcohol abuse	7 (23)	37 (12)	.16
Cluster B personality disorder	4 (13)	7 (2)	.01
ASD	9 (29)	28 (9)	.003
Autistic disorder or Asperger’s disorder	0 (0)	12 (4)	.61
PDD-NOS	9 (29)	16 (5)	.001

**Table 2Table 2:** **Comparisons between suicide attempters and non**-**attempters within ASD and non**-**ASD group**

	**ASD (** ***n*** **= 37)**			**Non-ASD (** ***n*** **= 299)**		
	**Suicide attempters (** ***n*** **= 9)**	**Suicide non-attempters (** ***n*** **= 28)**	***p***	**Suicide attempters (** ***n*** **= 22)**	**Suicide non-attempters (** ***n*** **= 277)**	***p***
Autism-Spectrum Quotient (AQ) mean (SD)	33.3 (6.9)	30.9 (6.5)	.58			
Age (year) mean (SD)	28.0 (10.9)	28.7 (8.2)	.87	38.8 (13.4)	43.1 (14.1)	.16
Range	19–55	18–51		20–62	18–83	
	*n* (%)	*n* (%)		*n* (%)	*n* (%)	
Female gender	6 (67)	14 (50)	.46	14 (64)	174 (63)	1
Already treated at the first visit	5 (56)	16 (57)	1	18 (82)	174 (63)	.10
Drug treatments at the first visit	5 (56)	15 (54)	1	14 (64)	156 (56)	.66
Anxiolytics	2 (22)	10 (36)	.69	8 (36)	99 (36)	1
Antidepressants	4 (44)	8 (29)	.43	7 (32)	92 (33)	1
Mood stabilizers	1 (11)	2 (7)	1	5 (23)	38 (14)	.34
Antipsychotics	0 (0)	4 (14)	.55	5 (23)	39 (14)	.34
Educational level			.12			.18
University or upper	3 (33)	19 (68)		6 (27)	118 (43)	
High school or lower	6 (67)	9 (31)		16 (73)	159 (57)	
Marital status			.37			.65
Married	3 (33)	5 (18)		12 (55)	177 (60)	
Unmarried, divorced, or widowed	6 (67)	23 (82)		10 (45)	110 (40)	
Employment			.40			.25
Employed, homemaker, undergraduate	8 (89)	20 (71)		12 (55)	186 (67)	
Unemployed or retired	1 (11)	8 (29)		10 (45)	91 (33)	
Living alone	1 (11)	7 (25)	.65	1 (5)	58 (21)	.09
Past suicidal behaviors	5 (56)	3 (11)	.01	11 (50)	38 (14)	<.01
Family history of psychiatric disorders	3 (33)	6 (21)	.66	6 (27)	71 (26)	.81
Family history of suicide	1 (11)	3 (11)	1	5 (23)	17 (6)	.02
Bipolarity	3 (33)	13 (46)	.70	10 (45)	104 (38)	.50
Agitation	8 (89)	3 (11)	<.01	10 (45)	34 (12)	<.01
Psychotic features	1 (11)	1 (4)	.43	3 (14)	16 (6)	.16
Physical diseases	0 (0)	1 (4)	1	3 (14)	48 (17)	1
Alcohol abuse	3 (33)	2 (7)	.08	4 (18)	35 (13)	.51
Cluster B personality disorder	1 (11)	2 (7)	1	3 (14)	5 (2)	.02
Subtypes of ASD			.02			
Autistic disorder or Asperger’s disorder	0 (0)	12 (43)				
PDD-NOS	9 (100)	16 (57)

**Table 3:** **Adjusted odds ratios of six significant variables for suicide attempts from Table** 1

	***β*** **(S.E.)**	**Wald**	**Adjusted OR**	**95%** **CI**	***p***
Agitation	1.97 (.46)	18.01	7.15	2.88–17.74	<.001
Past suicidal behaviors	1.46 (.48)	9.49	4.32	1.70–10.98	.002
PDD-NOS	1.40 (.62)	5.11	4.04	1.20–13.54	.024
Family history of suicide	1.17 (.62)	3.51	3.22	0.95–10.95	.061
Age under 29 years old	0.75 (.49)	2.35	2.11	0.81–5.50	.125
Cluster B personality disorder	0.91 (.87)	1.11	2.49	0.46–13.58	.292

Adjusted for gender; *β* standardized coefficient, *S.E*. standard error, *OR* odds ratio, *CI* confidence interval, *PDD*-*NOS* pervasive developmental disorder-not otherwise specified.

SPSS 19.0 for Windows (SPSS, Tokyo, Japan) was used for the statistical analyses. XLSTAT version 2014.1 (Addinsoft, Tokyo, Japan) was used for the receiver operating characteristic curve analysis. A two-tailed *p* value of less than 0.05 was regarded as statistically significant for all analyses.

### Ethics

We informed the patients of the anonymous inclusion in this retrospective study together with information that the study was approved by the Ethics Committee of the University of the Ryukyus, Japan. These statements also included the sentence that the participants could withdraw from this study without any penalty or loss of benefits for their treatments, although no patients refused to participate in this study.

## Results

### Profiles of ASD subjects

Among the 336 adult depressed patients, 37 (11%) were diagnosed with ASD. In these 37 patients, 25 (68%) were diagnosed with PDD-NOS while 9 (24%) were diagnosed with AS and 3 (8%) with autistic disorder. There was no significant difference in the proportion of females between ASD (54%) and non-ASD subjects (63%). The proportion of suicide attempters was significantly higher in ASD subjects (*n* = 9, 24.3%) than in non-ASD subjects (*n* = 22, 7.4%) (*p* <0.01). The mean score (±SD) of total AQ was 31.4 (±6.5) in the ASD patients. No significant difference in the AQ scores was observed between suicide attempters and non-attempters among ASD patients.

### Comparisons between suicide attempters and non-attempters

Table 1 summarizes comparisons of possible risk factors for suicide attempts between subjects with suicide attempts and those without among the depressed first-visit patients. The mean age was significantly younger in suicide attempters than in non-attempters (*p* = 0.02), and the percentage of subjects under 29 years was higher in suicide attempters that in non-attempters (*p* <0.01). The proportions of past suicidal behaviors (*p* <0.01), family history of suicide (*p* = 0.02), agitation during a depressive episode (*p* <0.01), comorbid cluster B personality disorder (*p* = 0.01), and comorbid ASD (*p* = 0.003) were all greater in suicide attempters than in non-attempters. The proportions of PDD-NOS in suicide attempters and non-attempters also differed significantly (*p* = 0.001).

### Contributing factors to suicide attempts

When ASD was initially evaluated for the binary logistic regression analysis as an independent variable to extract the contributing factors to suicide attempts, the contribution of ASD did not reach a significant level (odds ratio (OR) = 1.89, 95% confidence interval (CI) = 0.58–6.12). Based on the finding that suicidal attempters with ASD were all diagnosed with PDD-NOS (Table 1), PDD-NOS instead of ASD was used as an independent variable. As a result, agitation during a depressive episode (*p* <0.001), past suicidal behaviors (*p* = 0.002), and comorbid PDD-NOS (*p* = 0.024) were all significantly associated with suicide attempts (Table 3).

### Comparisons between suicide attempters and non-attempters within ASD and non-ASD group

The differences between suicide attempters and non-attempters within ASD and non-ASD group are summarized in Table 2. In the ASD group, the proportions of agitation during a depressive episode (*p* <0.01), past suicidal behavior (*p* = 0.01), and comorbid PDD-NOS (*p* = 0.02) were greater in suicide attempters than in non-attempters. In the non-ASD group, the incidence of past suicidal behaviors (*p* <0.01), family history of suicide (*p* = 0.02), agitation (*p* <0.01), and the proportion of comorbid cluster B personality disorder (*p* = 0.02) were higher in suicide attempters than in non-attempters.

### Comparison between methods of suicide attempts in patients with ASD and without ASD

**Figure 1:**
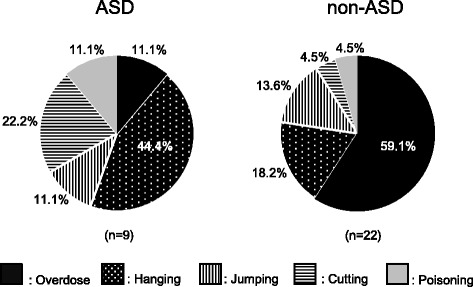
**Methods for suicide attempts in adult depressed patients with ASD and without ASD (non-ASD).**

Among the 31 suicide attempters, suicide methods were drug overdose in 14 cases (45%), followed by hanging in 8 cases (26%), jumping in 4 cases (13%), serious injury by cutting in 3 cases (10%), and poisoning in 2 cases (6%). Figure 1 shows that the most prevalent suicide method was drug overdose among non-ASD attempters while hanging was the most prevalent in ASD attempters.

## Discussion

The current results revealed that comorbid PDD-NOS, which includes atypical autism [1], was a significant risk factor for suicide attempts among first-visit patients with a major depressive episode together with other classical risk factors such as past suicidal behaviors and agitation during a depressive episode (Table 3).

It is not rare for clinicians to encounter patients with potential autistic traits among depressed adults in clinical settings. In fact, 11% of adult depressed patients or 29% of suicide attempters were finally diagnosed with ASD in the present study. Previous studies have also shown that ASD is not a negligible psychiatric diagnosis among suicide attempters, i.e., 7.3% in adults [14] and 12.8% in adolescents [42]. In addition, several studies point out that the prevalence of comorbid depression and anxiety is higher in individuals with ASD than in those without ASD [43-45]. These findings imply that there are close relationships between depression, suicidality, and autistic traits.

The major subtype of ASD was PDD-NOS (68%) in the present study, although most of these patients were not aware of their atypical and mild autistic traits at their first visit to our clinic. This is consistent with a previous study reporting that the majority of youth with ASD were diagnosed with PDD-NOS (88.5%) in a psychiatrically referred population [46]. Snow and Lecavalier [47] reported that individuals with PDD-NOS have more psychiatric symptoms such as anxiety and depression than those with autistic disorder. In addition, ASD subjects suffering from depressive symptoms show less social impairment and higher cognitive ability than those without depressive symptoms [48]. Moreover, there is a negative correlation between severity of ASD symptoms and internalizing problems [49] or levels of suicidal ideation [50]. Besides, PDD-NOS is not only a milder form of ASD but also has heterogeneous features, i.e., social and communication impairment without repetitive and stereotyped behaviors [51,52]. Some studies have indicated that many patients with high-functioning variants of ASD often stay unrecognized until late in adult life [53,54]. For these reasons, a high rate of PDD-NOS among adult depressed patients with ASD presented as a clinical reality in the current study. From another viewpoint, it is important for clinicians to know that the major manifestation of comorbid autistic traits is usually mild and atypical but sometimes accompanies suicidal risk in real-world clinical settings.

It was a striking result that all of the suicide attempters with ASD were diagnosed with PDD-NOS in the present study (Table 1). It is unclear why the “atypicality” of autistic traits contributes to the increased risk of suicide attempts. It is assumed that PDD-NOS individuals without awareness of the disorder or knowledge of the coping strategy may have been suffering from interpersonal problems, experience of isolation, and maladaptation to society for a long time since their childhood. This may lead to lowered self-esteem and failure in accessing support from others even after becoming adults [55]. Cognitive inflexibility, emotional dysregulation [56,57], and difficulty in identifying distressed feelings [58] may further provoke suicidal ideation or behaviors in these subjects. Thus, individuals with atypical and mild autistic traits paradoxically may be at higher risk for suicidality, which is exacerbated by the long-term preserved hopelessness and helplessness and the unresolved discrepancy between high demands for social adjustment and the low social and interpersonal skills.

In the present study, agitation was the most significant contributor to suicidal attempts among all the depressed adults (Table 3), and its incidence was extremely high (89%) among ASD suicide attempters (Table 2). These results suggest that agitation is a potent promoter for suicidal action in individuals with ASD. Although there have been few studies directly focusing on the relationship between agitation and suicide in ASD, some studies showed that agitation was one of the important manifestations among ASD patients visiting emergency departments [59,60]. Considering this fact together with the poor capability for cognitive and emotional control in ASD individuals [56,57], regular management and treatment of agitation may be important in reducing the potential risk of suicide attempts in ASD individuals. It is also important to advise that agitated ASD subjects with suicidal ideation are more prone to actually attempting or committing suicide.

Few studies have focused on the difference in the methods of suicide attempts between ASD and non-ASD subjects although it represents another important aspect for the risk assessment of suicidality. Kato et al. suggested that individuals with ASD tended to choose more lethal methods because of their less active imagination and lower impulsive control than the general population [14]. The same authors also demonstrated that suicide attempters with ASD required longer stays at the hospital or in intensive care as a result. In the present study, most of the non-ASD suicide attempters (59.1%) selected drug overdose as a less lethal and more uncertain suicide method (Figure 1). In contrast, almost a half of the ASD suicide attempters (44.4%) chose hanging, which has a higher lethality. Therefore, suicidality in depressed ASD patients should be considered cautiously from the aspect of frequency but also mortality.

The present study has some limitations. First, due to the retrospective study design, we could not completely assess all the subjects because the collected data was incomplete. Therefore, future studies should employ a prospective study design. Second, our ASD subjects were diagnosed using the older classification according to the DSM-IV-TR due to the study period ranging from 2009 to 2012. Therefore, our results should be confirmed by future prospective studies using the newer DSM-5. Third, subjects were recruited from first-time visitors to our outpatient clinic in a typical clinical setting. Therefore, there was an apparent imbalance in the number of subjects between suicide attempters and non-attempters due to the naturalistic study design. In addition, it should be noted that the profiles of suicide attempters in the present study might have been different from those of more risky suicide attempters being treated in psychiatric emergency care. Fourth, we did not assess the severity of depression and intelligence by adequate assessment tools.

## Conclusions

The present study suggests that comorbid PDD-NOS, rather than atypical autistic traits, are an important risk factor for suicide attempts among adult depressed patients in an outpatient clinical setting. Agitation is a potent promoter for suicidal actions in individuals with ASD. These subjects may also use more lethal methods for their suicide attempts than those without ASD.
